# A novel de novo *TBX5* mutation in a patient with Holt–Oram syndrome leading to a dramatically reduced biological function

**DOI:** 10.1002/mgg3.234

**Published:** 2016-07-14

**Authors:** Martina Dreßen, Harald Lahm, Armin Lahm, Klaudia Wolf, Stefanie Doppler, Marcus‐André Deutsch, Julie Cleuziou, Jelena Pabst von Ohain, Patric Schön, Peter Ewert, Ivan Malcic, Rüdiger Lange, Markus Krane

**Affiliations:** ^1^ Department of Cardiovascular SurgeryDivision of Experimental SurgeryGerman Heart Center Munich at the Technical University of MunichMunichGermany; ^2^ Bioinformatics Project SupportRomeItaly; ^3^ Department of Paediatric Cardiology and Congenital Heart DefectsGerman Heart Center Munich at the Technical University of MunichMunichGermany; ^4^ Department of PediatricsDivision of Cardiology and Intensive Care UnitUniversity Hospital ZagrebZagrebCroatia; ^5^ DZHK (German Center for Cardiovascular Research) – partner site Munich Heart AllianceMunichGermany

**Keywords:** Congenital heart disease, de novo mutation, heart‐hand syndrome, Holt–Oram syndrome, loss‐of function, TBX5, transcription factor

## Abstract

**Background:**

The Holt–Oram syndrome (HOS) is an autosomal dominant disorder affecting 1/100.000 live births. It is defined by upper limb anomalies and congenital heart defects with variable severity. We describe a dramatic phenotype of a male, 15‐month‐old patient being investigated for strict diagnostic criteria of HOS.

**Methods and results:**

Genetic analysis revealed a so far unpublished *TBX5* mutation, which occurs de novo in the patient with healthy parents. *TBX5* belongs to the large family of T‐box transcription factors playing major roles in morphogenesis and cell‐type specification. The mutation located in the DNA‐binding domain at position 920 (C→A) leads to an amino acid change at position 85 (proline → threonine). Three‐dimensional analysis of the protein structure predicted a *cis* to *trans* change in the respective peptide bond, thereby probably provoking major conformational and functional alterations of the protein. The p.Pro85Thr mutation showed a dramatically reduced activation (97%) of the *NPPA* promoter in luciferase assays and failed to induce *NPPA* expression in HEK 293 cells compared to wild‐type TBX5 protein. The mutation did not interfere with the nuclear localization of the protein.

**Conclusion:**

These results suggest that the dramatic functional alteration of the p.Pro85Thr mutation leads to the distinctive phenotype of the patient.

## Introduction

The Holt–Oram syndrome (HOS) is an autosomal dominant disorder affecting 1/100.000 live births. The syndrome was first described by Mary Holt and Samuel Oram (Holt and Oram 1960). The syndrome is assigned to the heart‐hand syndromes and is characterized by upper limb malformations and congenital heart disease (CHD) (Holt and Oram 1960; Basson et al. 1994, 1997, 1999; Li et al. 1997). Diagnosis of HOS should be followed by strict diagnostic criteria for HOS comprising the presence of preaxial radial ray malformation of at least one upper limb along with a personal or a family history of septation defects and/or atrioventricular conduction defects (McDermott et al. 2005). With these strict criteria around 70% of the HOS patients show a mutation in the *TBX5* gene (OMIM #601620) (Lichiardopol et al. 2007). Forty percent of HOS occur sporadically while the others are inherited by one of the parents (Holt and Oram 1960). In the beginning, mutations within the *TBX5* gene not related to HOS have not been identified (Goldmuntz 2004). However, it became evident that *TBX5* mutations are also associated with nonsyndromic diseases such as atrial fibrillation (Postma et al. 2008; Baban et al. 2014; Guo et al. 2016; Ma et al. 2016) or dilated cardiomyopathy (Zhang et al. 2015).

The correlation of the *TBX5* genotype with the severity of the clinical features of HOS remains a controversial issue. The majority of *TBX5* mutations cause both severe cardiac and skeletal phenotypes. Missense mutations lead to a large phenotype variation in the affected heart and limb depending on the position within the DNA‐binding domain. It has been suggested that missense mutations at the amino terminus of the DNA‐binding domain cause severe cardiac but milder skeletal abnormalities. On the other hand, mutations at the C‐terminus of the *TBX5* gene are responsible for milder cardiac defects but severe skeletal malformations (Basson et al. 1999). However, this relation has been questioned when a larger number of mutations and individuals were analyzed. Only two out of 20 individuals showed a clinical HOS phenotype consistent with the above mentioned prediction and even an identical *TBX5* mutation provoked a diverse clinical picture in first degree relatives (Brassington et al. 2003). Thus, the parameters and factors determining the genotype–phenotype relationship in HOS still have to be identified.

Upper limb defects are mostly bilateral, asymmetric and concern the preaxial radial ray. This includes predominantly thumb abnormalities with enormous phenotype variations (Poznanski et al. 1970; Basson et al. 1994; Newbury‐Ecob et al. 1996). Furthermore, the left side is more significantly affected (Smith et al. 1979). Most observed congenital heart defects are atrial septal defects (ASDs), ventricular septal defects (VSDs) and defects of the cardiac conduction system (Basson et al. 1997).

The main cause for HOS are mutations in the *TBX5* gene located on chromosome 12q24. Members of the T‐box gene family contain a highly conserved DNA‐binding motif of 180–190 amino acid residues (Simon 1999; Murray 2001; Tada and Smith 2001). T‐box genes are essential for cell‐type specification and morphogenesis (Smith 1999). Mutations in many of the T‐box genes are associated with human developmental disorders (Murray 2001). Examples are the CPX syndrome, caused by mutations in the *TBX22* gene (Braybrook et al. 2001), the DiGeorge syndrome (*TBX1* mutations) (Lindsay et al. 2001) and the ulnar‐mammary syndrome (*TBX3* mutations) (Bamshad et al. 1997). More than 90 mutations within the *TBX5* gene include nonsense, frameshift or splice‐site mutations, leading to dys‐functional proteins and resulting in haploinsufficiency are described (Basson et al. 1997; Kimura et al. 2015). Of these 90 mutations, more than 50 are missense mutations and deposited in the NCBI database (http://www.ncbi.nlm.nih.gov/SNP/). Furthermore, there are also case reports describing intragenic duplications (Patel et al. 2012) and translocations (Terrett et al. 1996) within the *TBX5* gene leading to HOS.

Here, we report a novel *TBX5* mutation within a highly conserved region of the TBOX domain which displays a severely reduced biological activity.

## Materials and Methods

### Ethical compliance

All donors have signed an informed consent and the procedure was approved by the local ethical committee of the medical faculty of the Technical University of Munich (Project number 5737/13).

### Patient and probands

The 15‐month‐old male patient was hospitalized with typical signs of the Holt–Oram syndrome in February 2014. He was the second child of healthy parents. His female sibling is three years older with a diagnosis of atrial septal defect II (ASD II). During the normal pregnancy there was no exposure to drugs, alcohol, smoking or infections. Birth occurred spontaneously with a weight of 3670 g and a length of 52 cm. Echocardiography and radiographs were carried out for the patient.

### Purification of genomic DNA from patient and first degree relatives

Peripheral venous blood samples were taken from the patient and both parents. Genomic DNA was purified using the QIAamp^®^ DNA Blood Mini kit according to the manufacturer′s recommendation (QIAGEN, Hilden, Germany).

### Amplification of genomic *TBX5* sequences by PCR and Sequencing

All eight coding exons of the *TBX5* gene (NM_000192.3) were amplified directly from genomic DNA by PCR, using the primers shown in the table (Table S1). Amplification of the genomic DNA fragments was carried out with the Fast Start High Fidelity PCR‐System (Roche Diagnostics, Mannheim, Germany) using 1x PCR reaction buffer containing 1.8 mm MgCl_2_, dNTPs (200 *μ* m each), 0.4 *μ* m of each primer, 5% DMSO and 1U Fast Start High Fidelity Enzyme Blend in a final volume of 20 *μ*L. A 4 min activation of the *Taq* polymerase at 95°C was followed by 40 cycles of 95°C for 30 sec, 60°C for 30 sec, 72°C for 120 sec and a final extension for 10 min at 72°C on a Biorad C1000^TM^ Thermal Cycler. Amplification of exon 5 and 9 was done using nested PCR. Therefore, 1 *μ*L of the first PCR reaction of exons 5 and 9 were amplified using the same conditions with the nested primers shown in the table (Table S1). PCR products were purified with the high pure PCR purification kit (Roche Diagnostics) and subjected to DNA sequencing at Source BioScience Imagenes (Berlin, Germany). Amplification of exon 4 was done in two independent PCRs and was sequenced in both cases. The sequences were blasted against the reference sequence of human *TBX5* (accession number NM_000192.3). The newly detected genetic variation has been submitted to the database of single‐nucleotide polymorphisms (db SNP) at NCBI (http://ncbi.nlm.nih.gov/SNP/).

### TBX5 protein structure and prediction of functional effects of the mutation

The coordinates of the TBX5–DNA complex were downloaded from the RCSB website (http://www.rcsb.org/pdb, (Berman et al. 2000), PDB identifier 2X6V) and pictures of the interaction between TBX5 and DNA have been generated using Discovery Studio Visualizer 4.5 (Accelrys Software Inc., San Diego, CA). The potential influence of the p.Pro85Thr mutation on the function has been assessed by PolyPhen‐2 v2.2.2r398 (http://genetics.bwh.harvard.edu/pph2/) and MutationTaster (http://www.mutationtaster.org/). The stability of the mutant protein was assessed using iStable (http://predictor.nchu.edu.tw/istable/).

### Expression plasmids and site‐directed mutagenesis

The p.Pro85Thr point mutation of the HOS patient was introduced into the wild‐type human *TBX5* sequence (fully sequenced, clone IRATp970D0542D, SourceBioscience, Nottingham, UK) in a pAW48 expression vector (backbone pcDNA3.1 with a flag sequence prior to the *TBX5* sequence, a kind gift by Dr. A. Moretti, Klinikum Rechts der Isar, Munich, Germany) by PCR‐based site‐directed mutagenesis, using the QuikChange Site‐directed Mutagenesis Kit (Stratagene, La Jolla, CA). The mutated primer 5′ GGCTGGAAGG CGGATGTTT**A**CCAGTTACAA AGTGAAGGTG 3′ (mutated base in bold) was used at a final concentration of 4 ng/*μ*L according to the manufacturer's instruction. After sequence verification of both the wild‐type and mutated *TBX5* sequences, the constructs were used in functional assays. Activity of the construct containing wild‐type or the mutated *TBX5* was tested in four independent luciferase assays.

### Nuclear localization of TBX5 protein

HEK 293 cells were grown to approximately 80% confluence on coverslides and transfected with wild‐type or mutated *TBX5* sequences (150 ng of plasmid) with FuGENE (Invitrogen, Darmstadt, Germany). Two days after transfection cells were subjected to immunocytochemistry. Cells were fixed with 4% paraformaldehyde for 15 min at room temperature and permeabilized with 0.1% Triton‐X‐100 in PBS for 10 min. Unspecific binding was blocked with 5% normal goat serum (abcam, ab7841, Cambridge, UK) for 30 min. Monoclonal mouse anti‐flag M2 (SIGMA, F1804, Munich, Germany) was diluted 1:1000 and rabbit polyclonal anti‐GAPDH IgG (abcam, ab36840) was diluted 1:200. HEK 293 cells were incubated with primary antibodies for 1 h. Secondary antibodies goat anti‐mouse IgG (H&L) Alexa‐Fluor 488 (abcam, ab150113) and goat anti‐rabbit IgG (H&L) Alexa Fluor 555 (abcam, ab150078) were used at a 1:500 dilution for 1 h. All incubations were performed at room temperature. After the last wash slides were air‐dried, mounted in Abcam mounting medium with DAPI (abcam, ab104139), sealed with cover slips and evaluated under a fluorescent microscope (Axiovert 200M, Zeiss, Oberkochen, Germany).

### Luciferase reporter assay

HEK 293 cells were cultured in DMEM/Ham′s F12 (Biochrome, Berlin, Germany) supplemented with 5% FCS (HyClone, Cramlington, UK) in a humidified atmosphere with 5% CO_2_. The reporter plasmid contains the *NPPA* promoter which drives the expression of luciferase (pALI‐Lva/ANF‐luciferase, a kind gift from Dr. Q. Wang, Cleveland, OH). HEK 293 cells were transfected with the pAW48 expression vectors together with the reporter plasmid using FuGENE HD (Invitrogen) according to the manufacturer′s recommendation. Each well received 150 ng of both effector and reporter plasmid and 250 ng of the pBluescript KSII plasmid which is not expressed in mammalian cells to normalize the DNA amount. Transfection efficiency in each well was normalized to a *β*‐galactosidase‐expressing construct (pCMV *β*‐Gal, added at 50 ng/well) in an ELISA. After two days of incubation cells were lysed in reporter lysis buffer (Promega, Madison, WI), incubated for 10 min at room temperature and frozen for at least 30 min at −80°C. Lysates were thawn, shaked for 30 min at room temperature and centrifuged for 2 min at 20.000 × *g*. Ten *μ*L of the supernatant were mixed with 100 *μ*L of substrate buffer and luciferase activity was measured in a luminometer (Tecan, Crailsheim, Germany). The measured activity of each sample was normalized to the *β*‐galactosidase expression of the sample. All samples have been tested in four independent experiments each performed in triplicates.

### Evaluation of transcriptional activity of wild‐type and mutant TBX5

HEK 293 cells were transiently transfected with the pAW48 expression vector harboring either wild‐type or mutated *TBX5* sequences. Two days later, cells were lysed with RNA lysis buffer (Peqlab, Erlangen, Germany). Total RNA was purified, using the peqGOLD total RNA kit (Peqlab) and reverse‐transcribed into cDNA with M‐MLV reverse transcriptase (Invitrogen). Expression of *NPPA* (NM_006172.3)*, TBX5* (NM_000192.3)*, β‐ACTIN* (NM_001101.3)*, CX40* (NM_005266.6), *IRX4* (NM_016358.2), *HEY2* (NM_012259.2)*, ID2* (NM_002166.4), and *TNNI3* (NM_000363.4) was evaluated on a LightCycler 1.5 (Roche Diagnostics) using the following conditions: activation of *Taq* polymerase for 15 min at 95°C followed by 40 cycles with 15 sec at 94°C, 20 sec at 60°C, and 20 sec at 60°C.

### Statistics

The significance of differences in luciferase activity was evaluated using the unpaired Student's *t*‐test. A value of *P *<* *0.05 was considered to be statistically significant.

## Results

### Identification of a novel de novo mutation p.Pro85Thr in exon 4 of the *TBX5* gene in a patient with Holt–Oram syndrome

**Figure 1:**
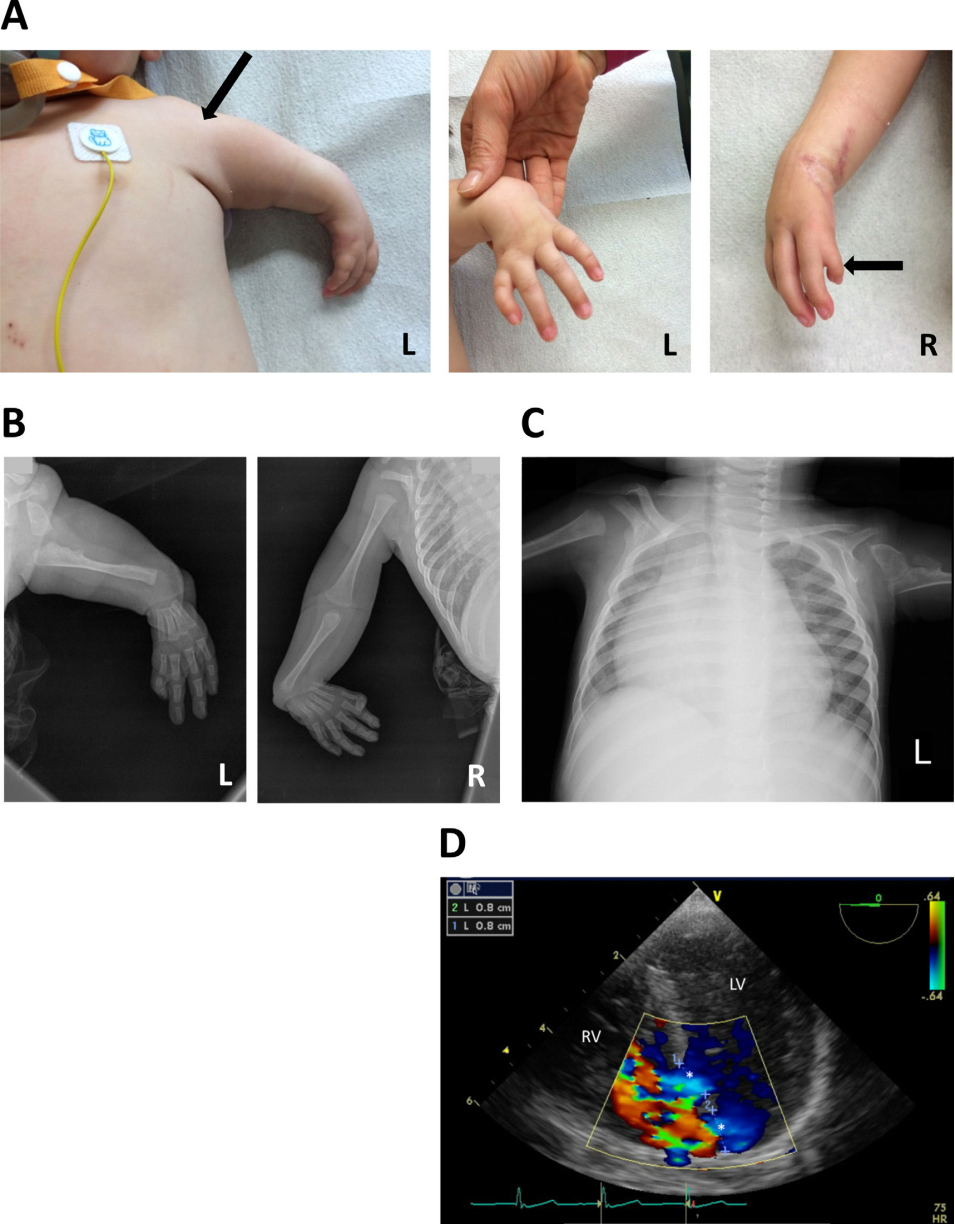
Upper limb anomalies and cardiac defects of the patient. (A) Photographs of the body and upper limbs showing the deformation of the upper arms and thumbs on both hands. (B) X‐rays of both upper limbs. (C) X‐ray of the body. (D) Echocardiogram showing the two VSDs (marked by *).

The 15‐month‐old male patient displayed pronounced and typical features of the Holt–Oram syndrome (HOS). The left upper arm was dramatically shortened (hypoplasia) and the thumb on this side was completely missing (agenesis). The right fore limb showed an aplasia of the radius and a deformed, shortened thumb (Fig. 1A). In addition, the mobility of both hands was reduced due to a radial flexion. X‐rays of the fore limbs confirmed these clinical findings and displayed the radial aplasia of the right fore limb and the malformation of the thumbs (Fig. 1B). Furthermore, the chest X‐ray indicated a severely enlarged heart, with a strongly enhanced cardiothoracic ratio of 0.75 (Fig. 1C). In addition, the patient showed distinct cardiac malformations including multiple VSDs, a patent foramen ovale (PFO) and a severe insufficiency of the tricuspid valve (grade III). Echocardiography clearly visualized the two VSDs with diameters of 0.8 cm each (Fig. 1D).

**Figure 2:**
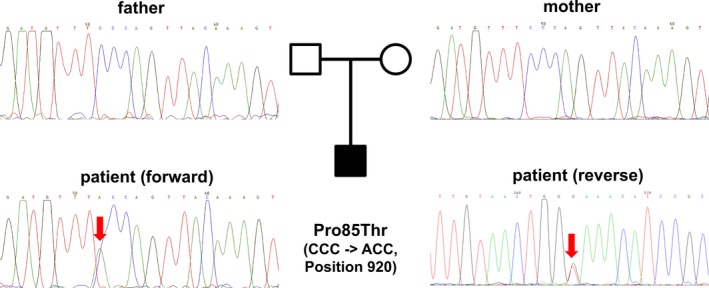
Identification of the p.Pro85Thr mutation in the *TBX5* gene. Chromatograms of exon 4 showing the sequences of the unaffected parents and the c.920_C>A mutation of the HOS patient. For the parents, the forward sequences are shown.

Due to the characteristic and extraordinary severe HOS phenotype we decided to sequence all coding exons of the *TBX5* gene directly from genomic DNA. In exon 4 we detected a single‐nucleotide change c.920_C>A leading to an amino acid change from proline to threonine at amino acid position 85 (Fig. 2). We then sequenced exon 4 of the *TBX5* gene in both parents who did not have any history of CHD or upper limb malformations. As expected exon 4 revealed a native wild‐type sequence in both cases (Fig. 2). Thus, the detected point mutation was not inherited but rather appeared de novo in the patient.

### Three‐dimensional structure of TBX5‐DNA interaction and conservation of the mutated residue

The de novo p.Pro85Thr mutation is located within the highly conserved TBOX domain which interacts with the DNA. This mutation has not yet been deposited in the NCBI dbSNP (www.ncbi.nlm.nih.gov/SNP) and the NHLBI Exome variant server (evs.gs.washington.edu/EVS) databases.

**Figure 3:**
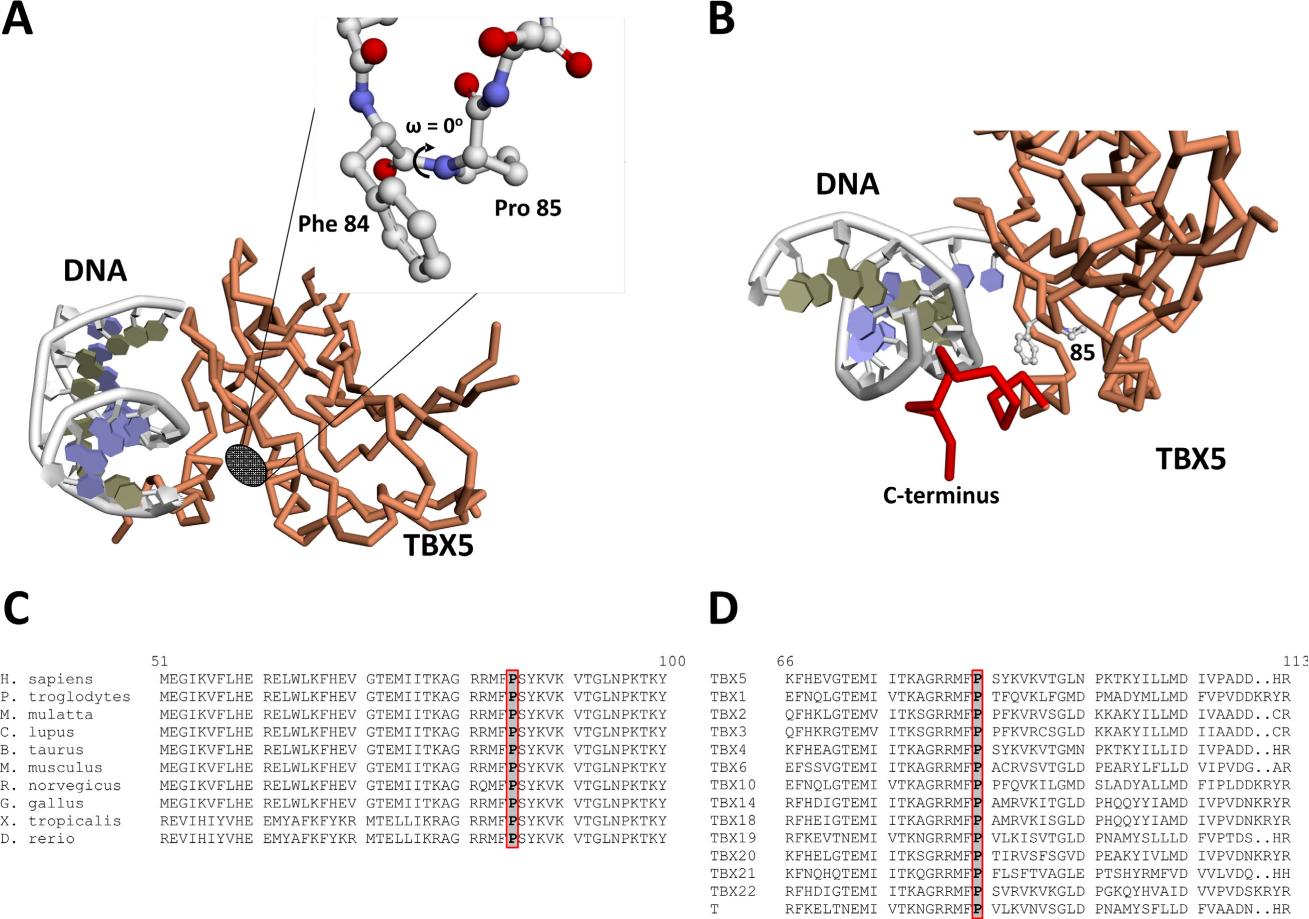
Location and conservation of the p.Pro85Thr mutation. (A) Schematic view of the interaction between TBX5 and DNA. The location of p.Pro85 and the *cis*‐peptide bond is indicated. (B) Vicinity of Phe84‐Pro85 and the TBX5 C‐terminus involved in DNA binding. (C) Alignment of human TBX5 protein sequence with TBX5 proteins of multiple species. (D) Alignment of human TBX5 protein with other members of the *T‐box* gene family. Numbers refer to amino acid positions of the human TBX5 sequence.

We first analyzed the potential effect of this mutation within the context of the TBX5‐DNA complex structure (Stirnimann et al. 2010) (Fig. 3A). In the wild‐type protein Phe84‐Pro85 adopts the rare *cis*‐peptide conformation (torsion angle ω = 0°) (Fig. 3B) occurring with a frequency of <0.3% in known protein structures (Weiss et al. 1998) with >90% of instances involving a proline residue (Xaa‐Pro, where Xaa might be any amino acid) (Weiss et al. 1998). A mutation into threonine at position 85 very likely adopts the usual *trans*‐conformation (torsion angle ω = 180°) leading to a local or even overall misfolding of the TBX5 protein compromising its proper function. Consistent with this, the iStable software package predicted a decrease in protein stability as a consequence of this specific mutation.

The p.Pro85Thr de novo mutation of the HOS patient is located within the DNA‐binding TBOX domain which spans amino acids 53 to 243 and is distal to the TBX5 C‐terminus (Fig. 3B), an essential element for binding the target DNA sequence (Stirnimann et al. 2010). Consequently, the most likely effect caused by the mutation will be impairment of proper DNA recognition and binding. An alignment of the human TBX5 protein sequence with the TBX5 sequences of other mammalian and non‐mammalian species revealed that the Pro85 residue is completely conserved during evolution (Fig. 3C). In addition, we compared the TBX5 protein sequence with the other members of the human T‐box gene family. Similarly, position Pro85 is absolutely conserved in all members (Fig. 3D). Finally, the mutation was estimated with a very high probability to be damaging by PolyPhen‐2 (score 1.000) and disease causing by MutationTaster (score 0.999).

### Effect of the p.Pro85Thr mutation on *NPPA* promoter activation

**Figure 4:**
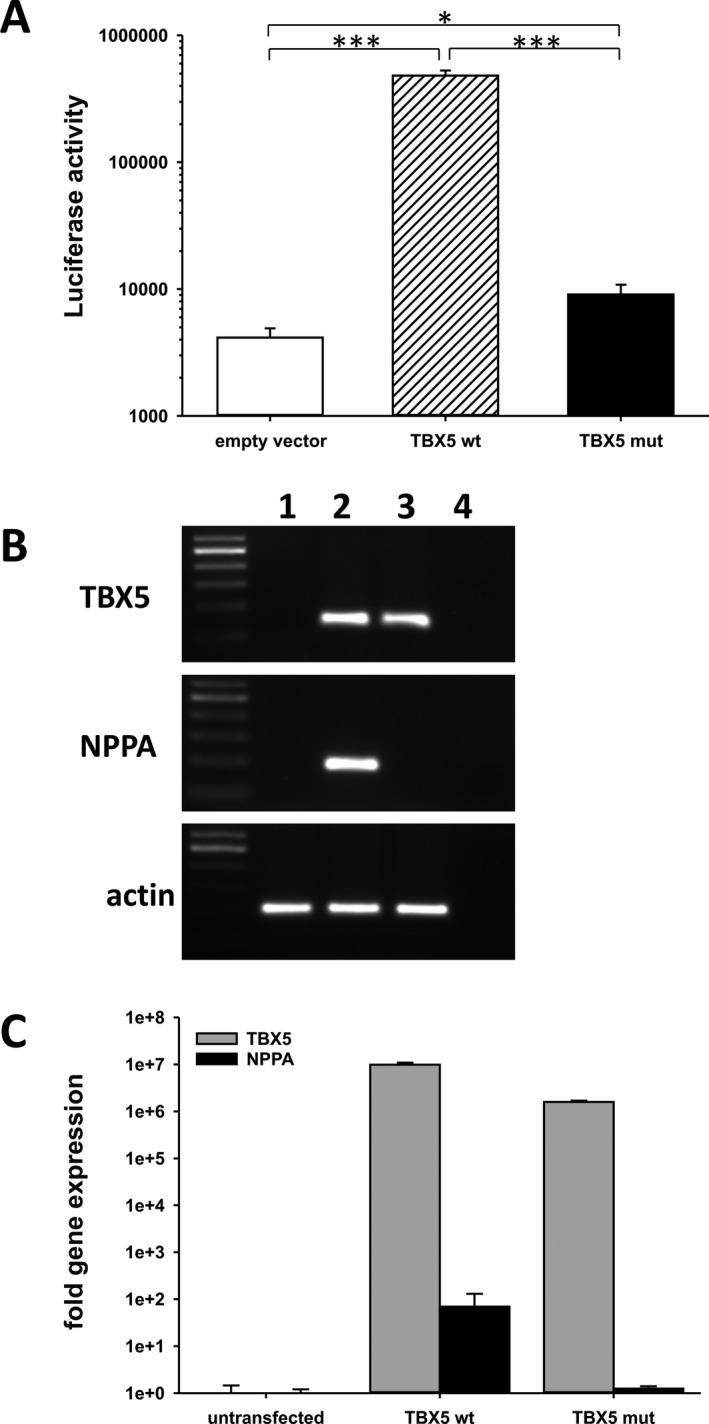
Functional analysis of the p.Pro85Thr mutant. (A) Activation of *NPPA* promoter‐driven luciferase activity in HEK 293 cells. Results are presented as the mean ± SE of four independent experiments. *P <* *0.05, ***P <* *0.001. (B) Induction of *NPPA* gene expression (NM_006172.3) in HEK 293 cells after transfection with wild‐type or mutant *TBX5* sequences. 1: untransfected, 2: wild‐type *TBX5*, 3: p.Pro85Thr *TBX5*, 4: aq.bidest. (C) qRT‐PCR analysis of *TBX5* (NM_000192.3) and *NPPA* expression in HEK293 cells. Fold gene expression was determined after normalization to an internal control (*β‐ACTIN,* NM_001101.3).

We tested the functional activity of the p.Pro85Thr mutant on the cardiac‐specific natriuretic peptide precursor type A (*NPPA*) promoter which drives the expression of the luciferase reporter gene. This promoter is well known to be activated by TBX5 (Hiroi et al. 2001). Indeed, wild‐type TBX5 was able to induce a strong, more than 100‐fold elevated response compared to the empty vector (Fig. 4A). The p.Pro85Thr mutant showed a dramatic, more than 95% reduced response, almost comparable to the activity of the empty vector (Fig. 4A).

### Effect of p.Pro85Thr on expression of *TBX5* target genes

We next addressed the potential of the p.Pro85Thr mutant to activate the *NPPA* promoter directly in HEK 293 cells which do not express endogenous *TBX5* (Fig. 4B). Upon transfection both the wild‐type and the mutant *TBX5* were equally expressed in these cells (Fig. 4B, C). Indeed, wild‐type TBX5 was able to induce *NPPA* gene expression while the p.Pro85Thr mutant failed to do so (Fig. 4B, C). Thus, the transcriptional activity of the mutant appears to be dramatically reduced compared to wild‐type TBX5. We have also evaluated the effect of the p.Pro85Thr mutant on the induction of further down‐stream targets like *CX40*,* IRX4*,* HEY2, ID2* and *TNNI3*. However, no effect was seen possibly due to the high endogenous expression of these genes in HEK 293 cells (Figure S1 and Table S2).

### Subcellular localization of the p.Pro85Thr mutant

**Figure 5:**
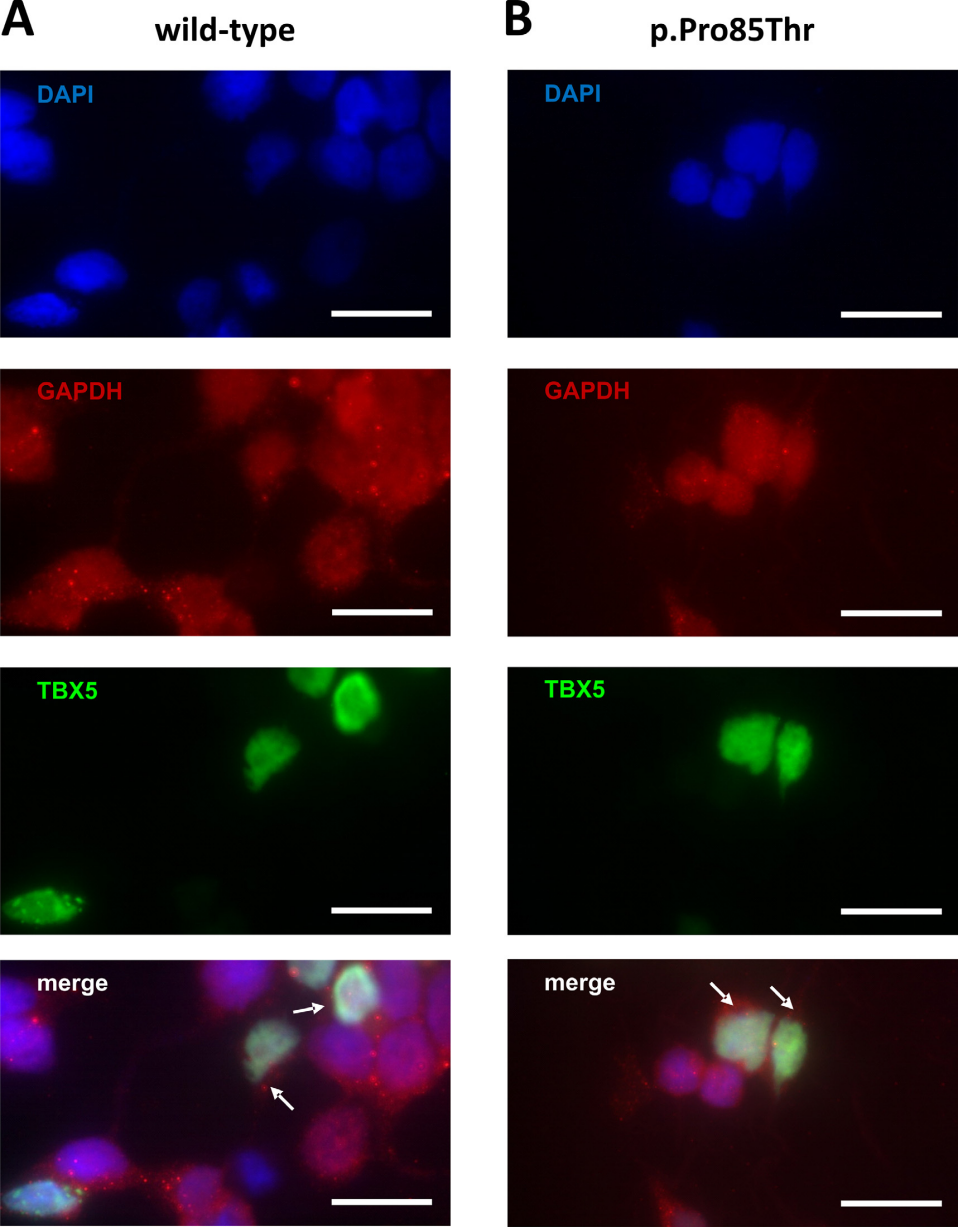
Nuclear localization of wild‐type (A) and mutant (B) TBX5 protein. Distribution of wild‐type and mutant TBX5 was analyzed by immunohistochemical staining using an anti‐flag antibody. Merged images show a combined staining of nuclei (blue), cytoplasm (red) and TBX5 (green). White arrows indicate cytoplasmic areas around the nuclei of TBX5 expressing cells. Scale bars represent 20 *μ* m.

To perform its function as a transcription factor, the TBX5 protein must enter the nucleus which is abolished by some *TBX5* mutations, at least in part (Fan et al. 2003). Therefore, we also compared the subcellular distribution of the p.Pro85Thr mutant with that of wild‐type TBX5 protein. To that end HEK 293 cells were transfected with expression vectors harboring flag‐tagged native or mutant *TBX5* sequences. To visualize the cytoplasmic compartment we performed an immunohistochemical staining with an anti‐GAPDH antibody. The distribution of wild‐type TBX5 was clearly restricted to the nucleus with no signs of cytoplasmic staining (Fig. 5A). Similarly, the p.Pro85Thr mutant also showed an exclusively nuclear localization (Fig. 5B), suggesting that the transport to the nucleus is not affected by the mutation. Thus, nuclear exclusion may not be a mechanism for the impaired biological activity of this specific mutation.

## Discussion

In the present paper we have identified a yet undescribed point mutation in the *TBX5* gene leading to an amino acid change at position 85 from proline to threonine. The diagnosis of HOS was made according to the strict diagnostic criteria (McDermott et al. 2005). The patient shows severe and asymmetric radial malformations in combination with multiple VSDs (“swiss cheese” VSD) and therefore complied with all strict diagnostic criteria for HOS. In approximately 70% of HOS patients the disease is associated with a mutation in the *TBX5* gene, including missense and nonsense mutations (Fan et al. 2003; Heinritz et al. 2005; Postma et al. 2008; Zhang et al. 2014; Al‐Qattan and Abou Al‐Shaar 2015; Guo et al. 2015) which are mostly located in the DNA‐binding TBOX domain (Mori and Bruneau 2004). The majority of these mutations are inherited while de novo *TBX5* mutations occur rather sporadically (Baban et al. 2014). The identified *TBX5* mutation was not inherited but rather occurred de novo as both parents displayed a wild‐type sequence.

The functional activity of TBX5 resides within the DNA‐binding TBOX domain which spans residues 52 to 243. The identified mutation is located in the N‐terminal region. Interaction studies using electrophoretic mobility shift assay experiments showed that the N‐terminal extension and the TBOX of TBX5 are essential for interaction with NKX2.5, whereas the C‐terminal domain is not (Hiroi et al. 2001). The identified mutation affects the proline 85 residue which adopts a rarely occurring *cis*‐peptide conformation (torsion angle ω = 0°), a main‐chain conformation which is nearly exclusively observed for proline residues combined with any other amino acid (Craveur et al. 2013). Specific isomerases generate and stabilize this high energetic structure (Schiene‐Fischer 2015). In the TBX5 protein of the HOS patient proline 85 was changed to a threonine residue which results in a switch to the common *trans*‐conformation of the peptide bond. This conformation alters the three‐dimensional structure of the mutated TBX5 protein around position 85, locally or even overall, likely affecting both folding and biological function. Therefore, also the interaction with other transcription factors to direct or cooperatively activate downstream targets may be influenced and thus may terminate in the dramatic phenotype of the patient. Most of the *TBX5* mutations result in a loss‐of‐function (Boogerd et al. 2010; Zhang et al. 2014; Kimura et al. 2015) and only a few of them show a gain‐of‐function (Postma et al. 2008; Baban et al. 2014). TBX5 is known to interact with promoter regions of several genes, including cardiac‐specific genes like *NPPA*. Therefore, we assessed the potential of the p.Pro85Thr mutation to activate the *NPPA* promoter which is a well‐known direct target of TBX5 (Hiroi et al. 2001). Wild‐type TBX5 protein strongly enhanced the activity of the *NPPA* promoter while the activation by the p.Pro85Thr was reduced by more than 95% suggesting a severe loss‐of‐function of the mutated TBX5 protein. This effect is similar though even more pronounced to that seen with other *TBX5* mutations located in that region which were also tested for *NPPA* promoter activation. These mutations include among others p.Gly80Arg (Ghosh et al. 2001; Hiroi et al. 2001), p.Met74Ile, pLeu94Arg (Boogerd et al. 2010) and p.Pro132Ser (Guo et al. 2016). In addition, the mutated p.Pro85Thr protein failed to induce the expression of the *NPPA* gene upon transfection of HEK 293 cells. Therefore, the mutation of *TBX5* at the amino acid position 85 appears to dramatically reduce the transcriptional activity. A regulation of the expression of other down‐stream targets like *CX40*,* IRX4*,* HEY2, ID2*, and *TNNI3* was not evident. This is most likely due to the fact that HEK 293 themselves express these genes abundantly. In addition, their regulation might require additional cofactors present in cells of the cardiac lineage but probably absent in HEK 293 cells.

Finally, we also explored nuclear exclusion as an additional mechanism to prevent proper transcriptional activity. Such a mechanism has been reported for other factors (Zhang et al. 2014) and some *TBX5* mutations also provoke a cytoplasmic localization of TBX5, at least in part (Fan et al. 2003). However, we did not see any difference of the subcellular localization between the wild‐type and the mutated protein. This is in good agreement with the results of Zaragoza and colleagues who have identified the nuclear localization signal of TBX5 at amino acid positions 325 to 327 (Zaragoza et al. 2004) well apart from position 85. They postulated the C‐terminal region following the TBOX domain harbors a transactivation domain, including a nuclear localization signal, which is far away from the mutated position in our patient.

In summary, we have identified a yet unknown mutation in the *TBX5* gene leading to an amino acid change at position 85 from proline to threonine. The mutation is located in a region which is highly conserved across species borders and all members of the T‐box family. The mutated protein displayed a dramatically reduced biological activity and failed to activate the promoter and expression of the *NPPA* gene. These severe functional defects may causatively lead to the pronounced clinical HOS phenotype of the patient.

## Conflict of Interest

All authors declare no conflict of interest.

## Supporting information


**Table S1** List of primers used.
**Table S2** qPCR analysis of relative expression of TBX5 downstream targets in HEK293 cells.
**Figure S1** Effect of wild‐type and p.Pro85Thr mutation on the expression of potential down‐stream targets.
