# Supplementary material for: A novel de novo TBX5 mutation in a patient with Holt–Oram syndrome leading to a dramatically reduced biological function
Source: Mol Genet Genomic Med. 2016 Jul 14;4(5):557–67. doi: 10.1002/mgg3.234 (PMC5023941; doi:10.1002/mgg3.234)
Supplement: Supplementary file 1 — Table S1 List of primers used. Table S2 qPCR analysis of relative expression of TBX5 downstream targets in HEK293 cells. Figure S1 Effect of wild‐type and p.Pro85Thr mutation on the expression of potential down‐stream targets. [file MGG3-4-557-s001.pdf]

Table S1. List of primers used.

target		sequence
**TBX5 Exon 2**	forward	CAC CCC CAT CTC CCC CTG
	reverse	GCC GAG CAG GAA AGC CAG A
**TBX5 Exon 3**	forward	GTC TCT GTG TTT TGG GGG AG
	reverse	CTC TTC CAA GCC ACC TTT TCT
**TBX5 Exon 4**	forward	TGG ATG GAG GCT GCC TTA AAA
	reverse	GTT CAC TGA TAC AAC TTT TCA AC
**TBX5 Exon 5**	forward	GGT GCG TGA ACT GAA GCA CG
	reverse	GGG AGA GAA ACC CAG TGA GA
**TBX5 Exon 5**	forward	TTC GGT GCA GTG CGC TAC C
**(nested)**	reverse	AGA GAG GAC AAG AGG GAG AC
**TBX5 Exon 6**	forward	AGC AGG GTT TTA TCT GGA GAC
	reverse	CAG CAG GAA AAC CTT GCA GAT
**TBX5 Exon 7**	forward	ATT AGC TCA TGT CCT GAG GTG
	reverse	GGT TGC TGC TGG CTT ACC TG
**TBX5 Exon 8**	forward	CCT GGT TCA GCC ACT CAG G
	reverse	TAC TCC TCA CCC CCT CAC C
**TBX5 Exon 9**	forward	TAA CTG TCT CCA CTT TTA GCT G
	reverse	GGG GTT CTC TTG GCT ACT GT
**TBX5 Exon 9**	forward	CCT GGT GCC TGC TCC TGG
**(nested)**	reverse	CTA CTG TCT CTC TCC TTC TCT
**b-ACTIN**	forward	CCA ACC GCG AGA AGA TGA
	reverse	CCA GAG GCG TAC AGG GAT AG
**TBX5**	forward	TGA TCA TAA CCA AGG CTG GA
	reverse	GAT TAA GGC CCG TCA CCT TC
**NPPA**	forward	GAG CGG ACT GGG CTG TAA C
	reverse	GGA GCC TCT TGC AGT CTG TC
**IRX4**	forward	CCT CAA GAG CTC CAA GAA CG
	reverse	CGA AGT CGT CCA AGT CAC TAA G
**CX40**	forward	CGT GGG CAG TTG GAG AAG
	reverse	GGG AAC AGA TGC CAA AAC TT
**HEY2**	forward	CTT GTC GCC TCT CCA CAA CT
	reverse	ATT CGG AGG GCT GAA TCC
**TNNI3**	forward	GCA GAT GCC ATG ATG CAG
	reverse	CAC CTC CCG GTT TTC CTT
**ID2**	forward	ATA TCA GCA TCC TGT CCT TGC
	reverse	AAA GAA ATC ATG AAC ACC GCT TA

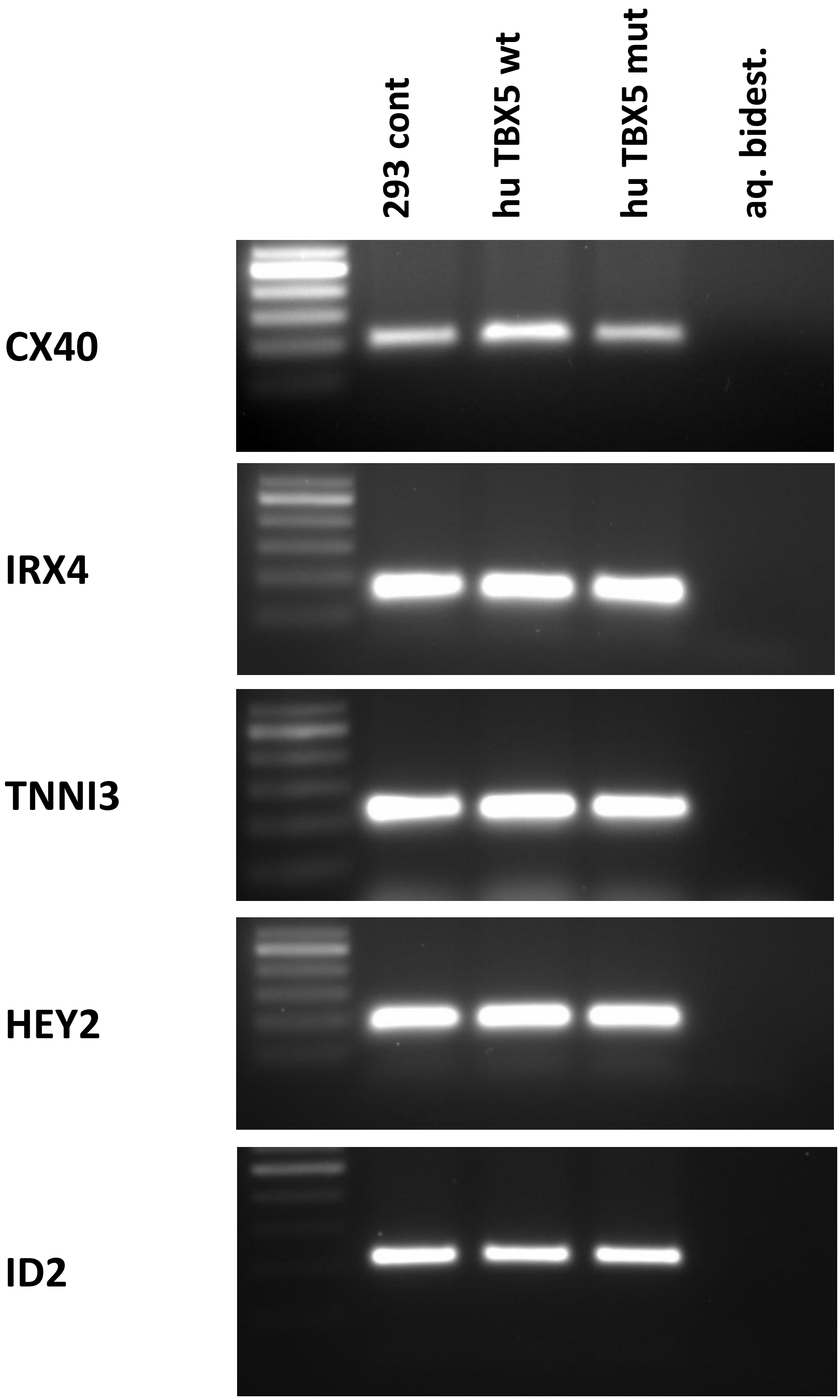

Figure S1. Effect of wild-type and p.Pro85Thr mutation on the expression of potential down-stream targets. HEK 293 were transfected with wild-type or mutant *TBX5* sequences and gene expression was analyzed by RT-PCR.

Table S2. qPCR analysis of relative expression of *TBX5* downstream targets in HEK293 cells

Gene	TBX5 wt	TBX5 mut
CX40	0.88 ± 0.34*	0.71 ± 0.05
IRX4	0.76 ± 0.19	1.24 ± 0.12
TNNI3	1.24 ± 0.03	1.14 ± 0.09
HEY2	1.42 ± 0.07	1.32 ± 0.02
ID2	0.93 ± 0.26	0.95 ± 0.03

: Values represent fold gene expression in HEK293 cells after transfection with wild-type (TBX5 wt) or p.Pro85Thr (TBX5 mut) sequences after normalization to an internal control (β-ACTIN*). The endogenous gene expression of untransfected HEK293 cells was arbitrarily set to 1.
